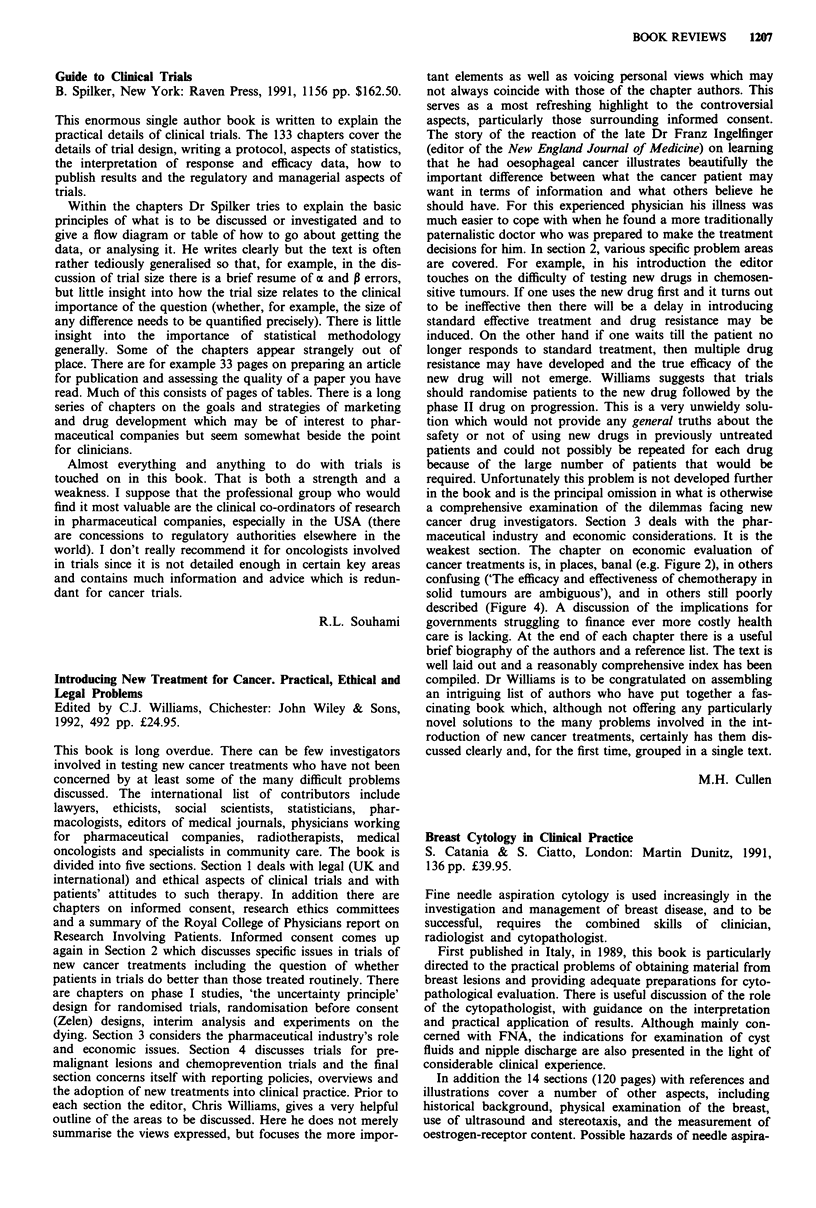# Introducing New Treatment for Cancer. Practical, Ethical and Legal Problems

**Published:** 1992-12

**Authors:** M.H. Cullen


**Introducing New Treatment for Cancer. Practical, Ethical and Legal Problems**
Edited by C.J. Williams, Chichester: John Wiley & Sons, 1992, 492 pp. £24.95.

This book is long overdue. There can be few investigators involved in testing new cancer treatments who have not been concerned by at least some of the many difficult problems discussed. The international list of contributors include lawyers, ethicists, social scientists, statisticians, pharmacologists, editors of medical journals, physicians working for pharmaceutical companies, radiotherapists, medical oncologists and specialists in community care. The book is divided into five sections. Section 1 deals with legal (UK and international) and ethical aspects of clinical trials and with patients' attitudes to such therapy. In addition there are chapters on informed consent, research ethics committees and a summary of the Royal College of Physicians report on Research Involving Patients. Informed consent comes up again in Section 2 which discusses specific issues in trials of new cancer treatments including the question of whether patients in trials do better than those treated routinely. There are chapters on phase I studies, 'the uncertainty principle' design for randomised trials, randomisation before consent (Zelen) designs, interim analysis and experiments on the dying. Section 3 considers the pharmaceutical industry's role and economic issues. Section 4 discusses trials for premalignant lesions and chemoprevention trials and the final section concerns itself with reporting policies, overviews and the adoption of new treatments into clinical practice. Prior to each section the editor, Chris Williams, gives a very helpful outline of the areas to be discussed. Here he does not merely summarise the views expressed, but focuses the more important elements as well as voicing personal views which may not always coincide with those of the chapter authors. This serves as a most refreshing highlight to the controversial aspects, particularly those surrounding informed consent. The story of the reaction of the late Dr Franz Ingelfinger (editor of the *New England Journal of Medicine*) on learning that he had oesophageal cancer illustrates beautifully the important difference between what the cancer patient may want in terms of information and what others believe he should have. For this experienced physician his illness was much easier to cope with when he found a more traditionally paternalistic doctor who was prepared to make the treatment decisions for him. In section 2, various specific problem areas are covered. For example, in his introduction the editor touches on the difficulty of testing new drugs in chemosensitive tumours. If one uses the new drug first and it turns out to be ineffective then there will be a delay in introducing standard effective treatment and drug resistance may be induced. On the other hand if one waits till the patient no longer responds to standard treatment, then multiple drug resistance may have developed and the true efficacy of the new drug will not emerge. Williams suggests that trials should randomise patients to the new drug followed by the phase II drug on progression. This is a very unwieldy solution which would not provide any *general* truths about the safety or not of using new drugs in previously untreated patients and could not possibly be repeated for each drug because of the large number of patients that would be required. Unfortunately this problem is not developed further in the book and is the principal omission in what is otherwise a comprehensive examination of the dilemmas facing new cancer drug investigators. Section 3 deals with the pharmaceutical industry and economic considerations. It is the weakest section. The chapter on economic evaluation of cancer treatments is, in places, banal (e.g. Figure 2), in others confusing ('The efficacy and effectiveness of chemotherapy in solid tumours are ambiguous'), and in others still poorly described (Figure 4). A discussion of the implications for governments struggling to finance ever more costly health care is lacking. At the end of each chapter there is a useful brief biography of the authors and a reference list. The text is well laid out and a reasonably comprehensive index has been compiled. Dr Williams is to be congratulated on assembling an intriguing list of authors who have put together a fascinating book which, although not offering any particularly novel solutions to the many problems involved in the introduction of new cancer treatments, certainly has them discussed clearly and, for the first time, grouped in a single text.

M.H. Cullen